# Tomato seedling stem and leaf segmentation method based on an improved ResNet architecture

**DOI:** 10.3389/fpls.2025.1571445

**Published:** 2025-08-28

**Authors:** Lina Zhang, Xinying Li, Zhiyin Yang, Bo Yang, Shengpeng Yu, Shuai Zhao, Ziyi Huang, Xingrui Zhang, Han Yang, Yixing Lin, Helong Yu, Minglai Yang

**Affiliations:** ^1^ College of Information Technology, Jilin Agricultural University, Changchun, China; ^2^ College of Information Engineering, Changchun University of Finance and Economics, Changchun, China

**Keywords:** plant phenotype, stem and leaf segmentation in point cloud, lightweight network, bottleneck block, downsampling

## Abstract

**Introduction:**

The phenotypic traits of tomato plants reflect their growth status, and investigating these characteristics can improve tomato production. Traditional deep learning models face challenges such as excessive parameters, high complexity, and susceptibility to overfitting in point cloud segmentation tasks. To address these limitations, this paper proposes a lightweight improved model based on the ResNet architecture.

**Methods:**

The proposed network optimizes the traditional residual block by integrating bottleneck modules and downsampling techniques. Additionally, by combining curvature features and geometric characteristics, we custom-designed specialized convolutional layers to enhance segmentation accuracy for tomato stem and leaf point clouds. The model further employs adaptive average pooling to improve generalization and robustness.

**Results:**

Experimental validation demonstrated that the optimized model achieved a training accuracy of 95.11%, a 3.26% improvement over the traditional ResNet18 model. Testing time was reduced to 4.02 seconds (25% faster than ResNet18’s 5.37 seconds). Phenotypic parameter extraction yielded high correlation with manual measurements, with coefficients of determination (R²) of 0.941 (plant height), 0.752 (stem diameter), 0.945 (leaf area), and 0.943 (leaf inclination angle). The root mean square errors (RMSE) were 0.506, 0.129, 0.980, and 3.619, respectively, while absolute percentage errors (APE) remained below 6% (1.965%–5.526%).

**Discussion:**

The proposed X-ResNet model exhibits superior segmentation performance, demonstrating high accuracy in phenotypic trait extraction. The strong correlations and low errors between extracted and manually measured data validate the feasibility of 3D point cloud technology for tomato phenotyping. This study provides a valuable benchmark for plant phenotyping research, with significant practical and theoretical implications.

## 1 Introduction

Tomato, as a crop of paramount importance globally (Ma et al., 2023), not only possesses significant economic value but is also rich in diverse nutrients, offering numerous health benefits to humans. In recent years, with the continuous advancement of agricultural technology, depth camera technology has demonstrated well application potential and significant technical advantages in the field of high-precision monitoring and analysis of plant phenotypic characteristics (Fang et al., 2023). This high-tech approach is capable of simultaneously capturing both the depth geometric structure information and detailed color texture features of plants. HeLi et al. pointed out that the phenotypic information of tea is an important phenotypic parameter to reflect the growth status of tea leaves and guide the management of tea garden (Li et al., 2022). Yixin Guo et al. pointed out that the stalk-related phenotype of soybean is important in soybean material selection (Guo et al., 2022). Peisen Yuan et al. show that in strawberry cultivation, phenotypic traits are decision tools for plant monitoring and management that can predict subsequent stages and key outcomes in plant development (Ndikumana et al., 2024). Through advanced algorithm processing, it can accurately reconstruct three-dimensional morphological models of plants. This capability has greatly enhanced the precision and depth of research on plant growth and development processes, providing comprehensive, reliable, and high-quality data support for scientific research work in fields such as crop breeding, and effectively promoting the in-depth development of plant science research.

The application of deep learning in the field of point cloud segmentation has become increasingly prevalent (Yang et al., 2024). Currently, the strategies for point cloud segmentation using deep learning methods can be mainly categorized into two types: those based on classical neural network architectures and those based on pre-trained neural network models. Models based on classical neural network architectures (Guo et al., 2020) primarily extract feature information from point cloud data, mapping these features into multiple subsets, where each subset corresponds to a specific feature dimension, and segmentation tasks are performed separately for each subset. Shuqi Fang et al. successfully achieved effective vehicle detection and precise segmentation by replacing the backbone network ResNet in the Mask R-CNN model with ResNeXt (Fang et al., 2023). Frans P. Boogaard et al. utilized a deep neural network based on PointNet to finely segment point clouds, thereby successfully estimating internode lengths from the three-dimensional point clouds of cucumber plants (Boogaard et al., 2023). JINHUI ZHANG et al. proposed an improved semantic segmentation network, RangeNet++, based on an asymmetric loss function. This network enhances point cloud segmentation performance by accurately calculating and adjusting target weights through the combination of an asymmetric loss function and the Adam optimizer (Zhang et al., 2023). Jingkun Yan et al. constructed a 3D deep learning network named PEPNet, which can accurately segment plant organs and extract stem and leaf phenotypic traits (Yan et al., 2024). Seunghan Yoon et al. proposed a VNet segmentation model that can rapidly annotate organs in CT images using SEED images, significantly improving annotation efficiency (Yoon et al., 2024). Muhammed Enes Atik et al. proposed a robust and efficient deep learning-based point cloud semantic segmentation method, which can accurately perform semantic segmentation on range images generated from spherically projected point clouds (Atik and Duran, 2022). Xianquan Han et al. designed a local multi-level feature fusion point cloud deep learning network and successfully applied it to segmentation tasks on two public datasets (Han et al., 2023). Xiaoguo Yang et al. proposed a new uncertainty-guided learning strategy (UGLS) to significantly enhance the ability of the U-Net neural network to segment multiple objects of interest from multi-modal images (Yang et al., 2024). However, it is worth noting that traditional neural network models often require a significant amount of computational resources and have longer training periods.

Based on pre-trained neural network architectures (Salehi et al., 2023), the pre-training process is executed on large-scale datasets, and the general features learned during this process can be transferred and applied to other specific tasks. Nanqing Dong proposed a strategy for pre-training the Region Proposal Network (RPN) within a multi-stage detector, along with a self-supervised learning strategy called ADePT. Experimental results indicate that the pre-training of RPN can significantly reduce its localization error (Dong et al., 2024). To fully utilize unlabeled data, Shoucun Chen et al. proposed a pre-training strategy based on contrastive learning, which can improve the accuracy of brain tumor labeling (Chen et al., 2022). Zihan Wang et al. constructed a multimodal pre-trained Transformer model for performing EEG-based DOC (Disorders of Consciousness) state classification tasks (Wang et al., 2024). Jiaao Li et al. proposed a novel framework called CLIPSP, along with an adaptive prompting method, aimed at leveraging the pre-trained knowledge of CLIP (Contrastive Language–Image Pre-training) for scene parsing (Li et al., 2024). Qing Ye et al. introduced GNPDTA (Graph Neural Network-based Predictive DTA) as a new method for DTA (Drug-Target Affinity) prediction, aiming to address the significant differences between the pre-training objectives and samples used in existing pre-training methods and the corresponding DTAP (Drug-Target Affinity Prediction) methods (Ye and Sun, 2024). Sung-Jin Kim et al. proposed a domain-agnostic Transformer model, named dformer, for generalizing EEG pre-training models (Kim et al., 2024). Zhaohu Xing et al. proposed a hybrid masked image modeling framework for pre-training in three-dimensional medical image segmentation, which supports both CNN (Convolutional Neural Networks) and Transformer structures, effectively extracting features from medical image data (Xing et al., 2024). However, it is worth noting that when using pre-trained neural network models for experiments, a large amount of data is usually required for training, which may lead to the occurrence of overfitting.

Compared to traditional two-dimensional representation methods, point cloud data (Rauch and Braml, 2023; Stilla and Xu, 2023) can more accurately capture the geometric shapes of objects and effectively depict their three-dimensional spatial structures, thereby demonstrating stronger representation capabilities when describing complex shapes and irregular objects. Jintao Chen et al. proposed a weakly supervised ALS point cloud semantic segmentation method based on line and plane point learning, and validated its effectiveness on three datasets (Chen et al., 2024). HAOXIANG SHI et al. introduced a self-supervised contrastive learning framework and incorporated few-shot contrastive learning with unsupervised data augmentation to enhance text clustering performance (Shi and Sakai, 2023). Kun Fang et al. designed a three-dimensional point cloud segmentation algorithm based on depth cameras, which is suitable for unsupervised class segmentation of large-scale model point clouds (Fang et al., 2023). Xinrong Bu et al. proposed a three-dimensional point cloud semantic segmentation network named DFSNet, which achieved good segmentation results in unstructured orchard sites (Bu et al., 2024). Xin Cao et al. introduced the PointStaClu method within the unsupervised learning framework to achieve single-stage point cloud clustering (Cao et al., 2024). Yinyin Peng et al. proposed a new self-distillation architecture for weakly supervised point cloud instance segmentation, which can utilize inaccurate bounding boxes as annotations for training (Peng et al., 2023). Muhammad Sulaiman et al. combined unsupervised segmentation techniques with a genetic algorithm-optimized combination method to validate the effectiveness of segmentation using LiDAR point cloud datasets (Sulaiman et al., 2024). Yongbin Liao et al. proposed the first semi-supervised point cloud instance segmentation network that uses bounding boxes as supervision, and this network can mine instance masks within predicted bounding boxes on both learned semantic score maps and original point clouds (Liao et al., 2021). However, it is worth noting that due to the limited label information provided before training in weakly supervised learning (Ren et al., 2023) and unsupervised learning (Ding et al., 2022), models require more resources during the training stage and may exhibit poor segmentation performance in some specific application scenarios.

Addressing the array of challenges currently faced in the field of point cloud segmentation, this paper presents the design and implementation of a lightweight point cloud segmentation network model, termed the X-ResNet network. The construction of this network model aims to effectively tackle the following key issues:

1. Conventional neural network models necessitate a substantial parameter during the training phase, accompanied by high computational complexity. This not only results in a prolonged training period and sluggish training speed but also leads to inefficiency, thereby compromising the performance of segmentation tasks.
2. When there exists a significant distribution discrepancy between the training data of a new model and an initial model, utilizing a pre-trained neural network model for training tends to readily induce overfitting, thereby weakening the generalization capability of the model and ultimately resulting in poor training outcomes.

Addressing the current issues, this paper adopts the following strategies:

1. Combined with the curvature features and geometric features to customize the convolution layer, improve the training speed of the model, make the convolution layer more fit to the point cloud data, and the model can better extract the stem and leaf data features of plants.
2. Integrated the encapsulated convolutional layer with downsampling operations, thereby enhancing the convergence speed and stability of the model during the training process.
3. By deeply integrating the traditional ResNet18 network with Bottleneck Blocks, the number of parameters and computational load of the model are reduced, thereby decreasing the model’s complexity. Taking tomato as an example, the proposed X-ResNet network successfully achieves high-throughput and precise extraction of plant phenotypic parameters.

## 2 Materials and methods

### 2.1 Data sources

**Figure 1:**
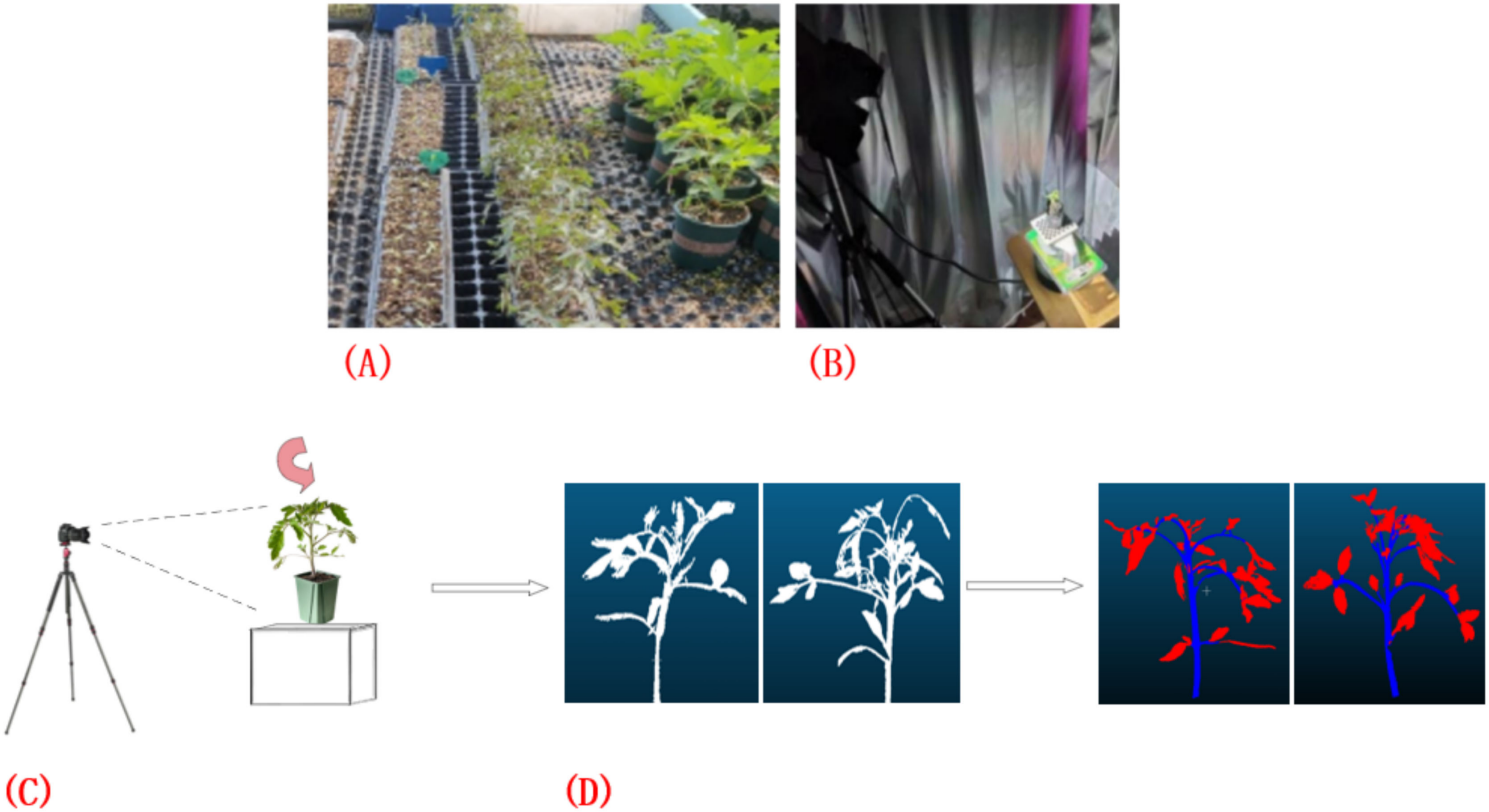
Point cloud data acquisition and annotation process. **(A)** Tomato plant sample point cloud data collection; **(B, C)** Acquisition of point cloud data using the 3DScanner-630w device; **(D)** Annotation of data using CloudCompare software.

This study was conducted within the No. 4 greenhouse facility of the Jilin Vegetable and Flower Science Research Institute, encompassing five distinct planting blocks, each with 96 plants cultivated, totaling 480 plant samples. In terms of planting layout, each region is divided into 2 rows with a distance between rows of 0.1 m and each line is 2.4 meters long (**Figure 1A**). Within the same row, the distance between adjacent plants was 0.05 meters. As shown in **Figures 1B, C**, the study employed the high-precision 3DScanner-630w (measurement dimension error: 0.001~0.03 mm; maximum lens pixel: 6.3 million; scanning mode: non-contact surface scanning; single plane scanning speed: about 1s; no limited scanning range) device to collect point cloud datasets of tomato plants. During the data collection process, special attention was paid to avoiding potential interference caused by shadow occlusion and surface reflection, to ensure comprehensive and accurate acquisition of the plant’s overall morphological information. Furthermore, as illustrated in **Figure 1D**, utilized the professional point cloud processing software CloudCompare to conduct detailed and accurate annotation of the collected data.

### 2.2 Algorithmic process

Captured the three-dimensional point cloud data of the plant using depth camera technology, which is typically represented as a set of three-dimensional coordinates P= ${\left\{{\text{p}}_{i}\right\}}_{i=1}^{N}$ ⊂ ${\text{R}}^{3}$ , where N denotes the total number of points in the point cloud, ${\text{p}}_{i}={\left[{\text{x}}_{i},{\text{y}}_{i},{\text{z}}_{i}\right]}^{T}$ . The preprocessing steps include the following common operations:

This research employed a statistical filtering approach (Lin et al., 2024) to process the neighborhood of the point cloud, aiming to remove noise points. Assuming that each point has a neighborhood N( ${\text{p}}_{\text{i}}$ ), it is calculated by Equation 1 by the following conditions.


(1) $${\text{Retain points p}}_{i}:\frac{1}{\left|\text{N}\left({\text{p}}_{i}\right)\right|}\sum_{\text{q}\in \text{N}\left({\text{p}}_{\text{i}}\right)}{\left\|{\text{p}}_{i}-\text{q}\right\|}_{2}^{2}\leq \in$$

Data normalization (Zhao et al., 2024) is a technique that maps data to a uniform scale or distribution range, aiming to enhance the efficiency and performance of machine learning algorithms, eliminate differences among feature dimensions, and optimize data visualization. For the extracted three-dimensional point cloud data, performed normalization to ensure that the center of the point cloud is located at the origin and that the distribution of the point cloud is within a unit sphere, calculated by the Equation 2.


(2) $${\text{p}}_{i}^{'}=\frac{{\text{P}}_{i}-\bar{P}}{{\text{max}}_{j}{\left|\left|{\text{P}}_{j}-\bar{P}\right|\right|}_{2}}$$

Where, $\bar{P}=\frac{1}{\text{N}}\sum_{\text{i}=1}^{N}{\text{P}}_{i}$ denotes the geometric centroid of the point cloud.

The ResNet network incorporates residual connections (Park et al., 2021), enabling each layer to directly “learn” the residual between the input and the desired output, thereby reducing the complexity of model training. In the ResNet18 network, the residual block is constructed with two convolutional layers, each followed by a Batch Normalization (BN) layer (Saeedi et al., 2023). The core function of the BN layer is to normalize each data sample flowing through it, which accelerates the convergence process of model training, enhances the training stability of the model. Furthermore, by normalizing the input distribution of each layer, the BN layer ensures the numerical stability of gradients during the backpropagation, thereby mitigating the issues of gradient vanishing and gradient explosion. Its output function can be expressed as Equation 3:


(3) $$\text{F}=\gamma \frac{x_{i}-\mu }{\sqrt{\sigma^{2}+\epsilon }}+\beta$$

Wherein, μ represents the sample mean, σ^2^ represents the sample variance, γ is the scaling parameter, β is the shift parameter, and ϵ is a very small value.

The encoder module (Chen and Guo, 2023) is responsible for converting the input point cloud data into feature representations with semantic information. Assuming the input point cloud data is denoted as $\text{P}\in {\text{R}}^{N\times 3}$ , the encoder contains a bottleneck block, whose specific structure can be defined as follows Equation 4:


(4) $${\text{F}}_{e}={\text{f}}_{enc}\left(\text{P}\right)$$

Wherein, ${\text{F}}_{e}\in {\text{R}}^{M\times C}$ represents the encoded features, M denotes the number of points after downsampling, and C signifies the feature dimension.

Within the realm of deep learning, the Bottleneck Block (Jabeen et al., 2024) represents a specially designed deep neural network structure aimed at reducing the computational cost and total number of parameters during model training, thereby achieving the dual objectives of lowering model complexity while maintaining training accuracy. The Bottleneck Block integrates two key components: convolution operations (Wei et al., 2023) and activation functions (Mao and Zhou, 2023).The calculation formula is shown in Equation 5.


(5) $${\text{F}}_{1}=\sigma \left(\text{P}\cdot {\text{W}}_{1}+{\text{b}}_{1}\right),{\text{F}}_{e}={\text{F}}_{1}\cdot {\text{W}}_{2}+{\text{b}}_{2}$$

Wherein, ${\text{W}}_{1}\in {\text{R}}^{3\times C_{1}}、{\text{W}}_{2}\in {\text{R}}^{C_{1}\times C}$ represents the weight matrix, and σ denotes the nonlinear activation function.

**Figure 2:**
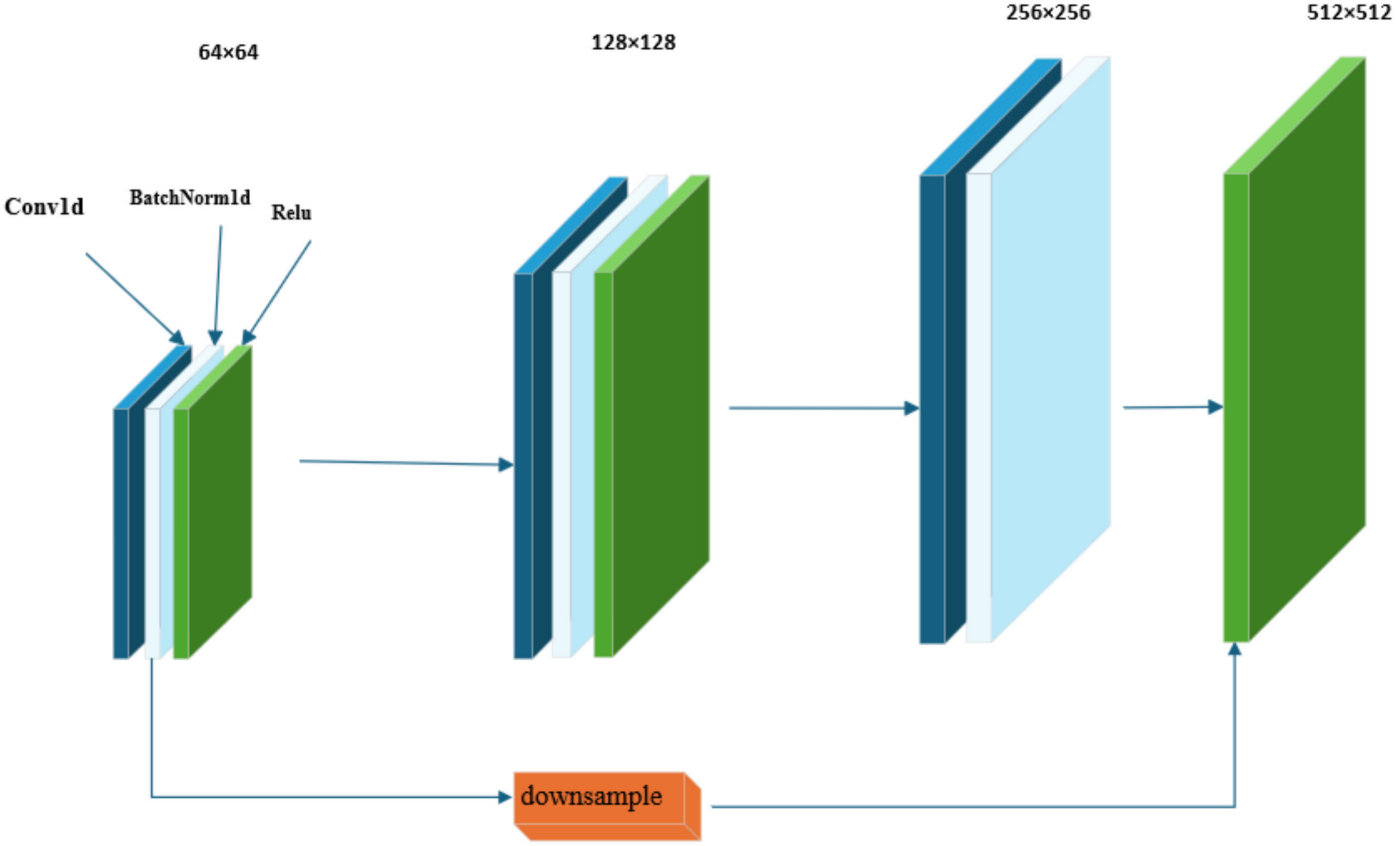
Bottleneck block architecture in the X-ResNet network framework.

As illustrated in **Figure 2**, within the framework of the residual structure of the ResNet18 network, the Bottleneck Block introduces a design of a 1×1 convolutional layer. The primary function of the first 1×1 convolutional layer is to reduce the number of channels in the feature map, thereby achieving a reduction in data dimensionality. The subsequent 3×3 convolutional layer is responsible for performing the core task of feature extraction. The last 1×1 convolutional layer restores the depth of the feature map by restoring the number of channels to the original output channels.

Given that the encoder component employs a specific transformation strategy, which efficiently maps the input raw data into a low-dimensional vector space, achieving a significant effect in data dimensionality reduction. In the decoder section, the model integrates three Bottleneck Blocks and directly introduces the encoder’s output into the decoder through skip connections for further processing (Kim and Lee, 2023). The definition of the decoder is now stated as follows Equation 6:


(6) $${\text{F}}_{d,i}={\text{f}}_{dec,i}\left({\text{F}}_{d,i-1},{\text{F}}_{e}\right)$$

Wherein, i=1, 2, 3 denote the indices of the respective Bottleneck Blocks, ${\text{F}}_{d,0}={\text{F}}_{e}$ . The final output feature of the decoder is labeled as ${\text{F}}_{d}\in {\text{R}}^{N\times C^{‘}}$ . The operation of the skip connection can be represented as Equation 7:


(7) $${\text{F}}_{d,i}=\text{Concat}\left({\text{F}}_{d,i-1},{\text{F}}_{e}\right)$$

Wherein, Concat is employed as a feature concatenation operation. Additionally, integrated downsampling techniques (Shen et al., 2023) into this treatment flowsheet. Downsampling gradually restores the data dimensionality to a level close to the original input data, it significantly enhances the model’s capability in feature extraction by reducing information redundancy and highlighting the saliency of key features.

By embedding downsampling operations within convolutional layers, this research are able to reduce the dimensionality and size of feature maps while extracting features from point cloud data. This approach decreases the complexity of the model, mitigates the risk of overfitting. In this experimental design, selected the Farthest Point Sampling (FPS) method as the downsampling strategy. The calculation formula is shown in Equation 8.


(8) $${\text{P}}_{sampled}=\text{Sample}\left({\text{P}}_{in},\text{r}\right)$$

Wherein, ${\text{P}}_{sampled}\in {\text{R}}^{M\times 3},\left(\text{M}<\text{N}\right)$ represents the number of points after sampling, and r denotes the sampling rate.

For feature processing, this research employ the k-Nearest Neighbors (Ni et al., 2024) pooling operation to aggregate neighborhood features. The calculation formula is shown in Equation 9.


(9) $${\text{F}}_{down}\left(\text{i},:\right)=\text{F}j\in N_{i}\max up\left(\text{j},:\right)$$

Herein, ${\text{N}}_{i}$ denotes the index set of neighboring points corresponding to the ith sampling point.

Assuming that the low-resolution features in the decoder are represented as ${\text{F}}_{low}\in {\text{R}}^{M\times C}$ , and the upsampling process aims to restore the features to the number of original points N. This process is typically achieved using methods such as Nearest Neighbor Interpolation or Trilinear Interpolation. The calculation formula is shown in Equation 10.


(10) $${\text{F}}_{up}\left(\text{i},:\right)=\sum_{j\in N_{i}}{\text{w}}_{ij}\cdot {\text{F}}_{low}\left(\text{j},:\right)$$

Herein, ${\text{w}}_{ij}$ represents the interpolation weights (which are usually determined by spatial distances, such as ${\text{w}}_{ij}=\frac{1}{\|{\text{P}}_{sampled,j}-{\text{P}}_{in,i}{\|}_{2}}$ ). ${\text{N}}_{i}$ represents the interpolation neighborhood for the i th original point.

The features thus restored are denoted as ${\text{F}}_{up}\in {\text{R}}^{N\times C}$ .

Adaptive Average Pooling (Wang et al., 2023) is a unique pooling mechanism characterized by its ability to handle input data of arbitrary sizes. This mechanism dynamically adjusts the size of the output data based on preset parameters to ensure strict matching with the input of subsequent fully connected layers in terms of size and dimension, thereby maintaining the coherence of the network structure and the consistency of data flow. In this hypothesis, the global feature ${\text{F}}_{global}\in {\text{R}}^{C}$ is obtained by calculating the average of the features of all points. The calculation formula is shown in Equation 11.


(11) $${\text{F}}_{global}\left(\text{c}\right)=\frac{1}{\text{N}}\sum_{i=1}^{N}{\text{F}}_{up}\left(\text{i},\text{c}\right)$$

Where c ∈ [1, C], The Adaptive Average Pooling operation adjusts the size of the feature map to (batch_size, 512, 1), a characteristic that significantly enhances the flexibility and compatibility of the network structure.

The Flatten layer (Zou et al., 2024) is responsible for flattening the multi-dimensional feature map output by the Adaptive Average Pooling layer into a one-dimensional vector, facilitating subsequent processing and analysis by fully connected layers. The calculation formula is shown in Equation 12.


(12) $${\text{F}}_{flat}={\left[{\text{F}}_{global}\left(1\right),{\text{F}}_{global}\left(2\right),\ldots ,{\text{F}}_{global}\left(\text{C}\right)\right]}^{T}\in {\text{R}}^{C}$$

The size of the feature map has been transformed to (batch_size, 512). Each neuron in the Fully Connected Layer establishes synaptic connections (Dong et al., 2023) with all neurons in the previous layer through full connectivity, and utilizes a unique set of weight parameters to achieve a nonlinear mapping from the high-dimensional feature space to the low-dimensional output space.

Assuming that the point cloud features after upsampling are denoted as ${\text{F}}_{up}\in {\text{R}}^{N\times C}$ , then mapped through a Fully Connected Layer to obtain the category distribution $\text{O}\in {\text{R}}^{N\times K}$ for each point, where K represents the total number of predefined categories. The calculation formula is shown in Equation 13.


(13) $$\text{O}\left(\text{i},:\right)=\text{Softmax}({\text{F}}_{up}\left(\text{i},:\right)\cdot {\text{W}}_{o}+{\text{b}}_{o})$$

Where:

${\text{W}}_{O}\in {\text{R}}^{C\times K}$ is the weight matrix,

${\text{b}}_{o}\in {\text{R}}^{K}$ is the bias vector,

$\text{Softmax}\left(\text{z}\right)=\frac{\text{exp}\left({\text{z}}_{k}\right)}{\sum_{k=1}^{K}\text{exp}\left({\text{z}}_{k}\right)}$ is the activation function used to generate the probability for each category.

Ultimately, the segmentation result comprises the category labels corresponding to each point. The calculation formula is shown in Equation 14.


(14) $${\hat{y}}_{\text{i}}={\text{arg}}_{k\in \left[1,K\right]}^{\;\max}\text{O}\left(\text{i},\text{k}\right)$$

Where ${\hat{y}}_{i}$ ∈{1,2,…,K} represents the predicted category for the i th point.

### 2.3 X-ResNet network model

**Figure 3:**
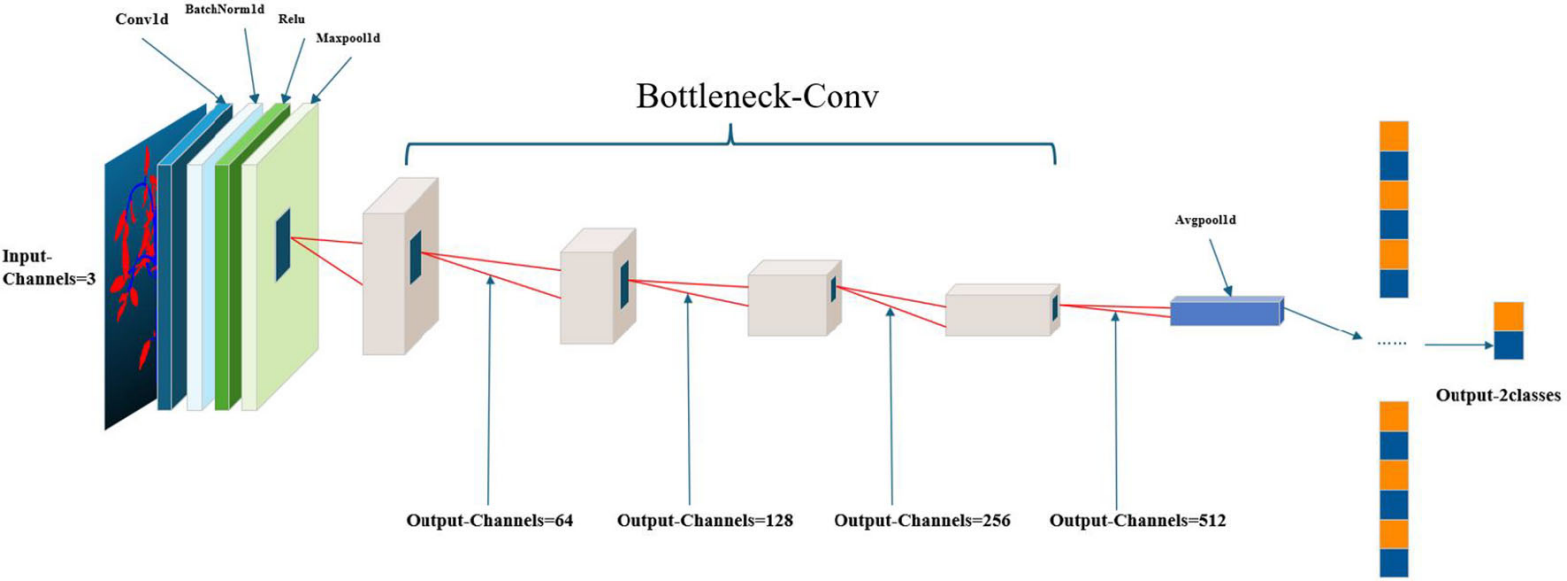
Diagram of the X-ResNet network architecture.

To enhance the quality of the input point cloud data, a series of preprocessing operations were executed. The architecture diagram of the X-ResNet network model is shown in **Figure 3**, using the custom-wrapped convolution layers, the proposed model architecture adheres to the encoder-decoder paradigm, wherein the encoder section comprises a bottleneck layer that conducts in-depth analysis of the input data, captured the core information within, and accordingly generates a feature vector rich in semantics. The decoder section is constituted by a cascade of three bottleneck layers employing skip connections, allowing the decoder to directly access and effectively integrate the feature information extracted by the encoder into its structure. Meanwhile, the decoder is responsible for restoring the low-resolution feature maps outputted by the encoder to the spatial resolution of the original data. The combined application of the encoder and decoder not only significantly reduces the data dimensionality but also effectively decreases the model complexity. During this process, an adaptive average pooling layer is first applied to adaptively reduce the spatial dimensions of the feature maps to a preset size, followed by a Flatten operation that flattens the reduced feature maps into a one-dimensional vector. Ultimately, this vector undergoes feature integration and output through a fully connected layer.

## 3 Results

### 3.1 Comparative experiment

In this experiment, accuracy, recall, precision, loss rate, F1 Score, and Intersection over Union (IoU) are employed as evaluation metrics to comprehensively assess the effectiveness of model training. The relevant calculation formulas are presented below.


$$\text{Accuracy}=\frac{\text{TP}+\text{TN}}{\text{TP}+\text{TN}+\text{FP}+\text{FN}}$$

$$\text{Recall}=\frac{\text{TP}}{\text{TP}+\text{FN}}$$

$$\text{Precision}=\frac{\text{TP}}{\text{TP}+\text{FP}}$$

$$\text{F}1\text{ Score}=2*\frac{\text{Precision}*\text{Recall}}{\text{Precision}+\text{Recall}}$$

$$\text{IoU}=\frac{{\text{S}}_{\text{Intersection}}}{{\text{S}}_{\text{Union}}}$$

Specifically, TP (True Positive) represents true positives, which is the number of samples correctly predicted as positive; FP (False Positive) represents false positives, which is the number of samples incorrectly predicted as positive when they are actually negative; FN (False Negative) represents false negatives, which is the number of samples incorrectly predicted as negative when they are actually positive; TN (True Negative) represents true negatives, which is the number of samples correctly predicted as negative.

**Figure 4:**
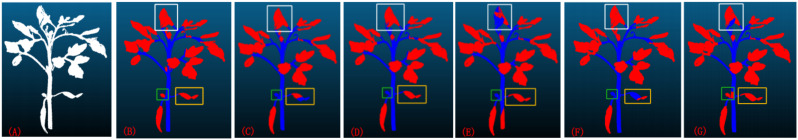
**(A)** the original image; **(B)** the segmentation results obtained after training using the X-ResNet network; **(C–G)** respectively display the segmentation results obtained after training using the ResNet18, PointNet, PointNet++, U-Net, and Mask R-CNN networks.

Currently, the mainstream deep learning models for point cloud segmentation encompass the ResNet18, PointNet, PointNet++, U-Net, and Mask R-CNN network models. To comprehensively evaluate the performance of the X-ResNet network model, conducted a detailed comparative analysis with the aforementioned five models serving as benchmarks. **Figure 4** visually presents the segmentation images obtained from training using six different network models. When the leaf morphology is elongated and curved, the leaves in **Figures 4C, 4F** are not fully recognized, reflecting the poor segmentation performance of the ResNet18 and U-Net network models in such cases; when the leaf area is too small, **Figures 4C–4F** all fail to identify the leaves, indicating that the ResNet18, PointNet, PointNet++, and U-Net network models fail to effectively capture leaf features during training, resulting in incomplete segmentation; when there is adhesion between leaves, the leaf contours in **Figures 4E, 4G** are not depicted clearly, further revealing the inadequate segmentation performance of the PointNet++ and Mask R-CNN network models in handling such complex situations. In contrast, when using the X-ResNet network model for training and segmentation operations, the segmentation effect of stems and leaves is the most ideal, and the contours of both are also extremely clear. This result fully demonstrates the superior performance advantages of the X-ResNet network model in plant stem and leaf segmentation tasks.

As demonstrated in **Table 1**, when compared to the ResNet18 network model, X-ResNet model exhibits an increase in accuracy by 3.26, recall by 9.96, precision by 4.43, and IoU by 3.43. Although the ResNet18 model achieves comparable accuracy during the training phase, it utilizes a larger number of parameters and exhibits higher model complexity. In contrast, the X-ResNet model reduces the parameter count by 59.6% compared to ResNet18, indicating its successful implementation of a lightweight design that mitigates model complexity.

**Table 1:** Performance evaluation of six network models (X-ResNet, ResNet18, PointNet, PointNet++, U-Net, and Mask R-CNN) for tomato plant image segmentation tasks.

Models	Accuracy (%)	Recall (%)	Precision (%)	Loss	F1-score (%)	IoU (%)	Params (M)
ResNet18	91.85	85.15	90.61	2.035	91.08	87.33	11.2
PointNet	87.39	83.45	83.66	3.810	84.42	81.92	8.02
PointNet++	86.74	87.35	88.93	3.785	83.71	81.99	7.66
U-Net	89.03	91.22	84.12	3.100	87.92	77.29	30
Mask R-CNN	87.62	85.23	85.35	3.247	84.75	86.93	20.6
X-ResNet	95.11	95.11	95.04	1.113	95.10	90.76	4.52

**Figure 5:**
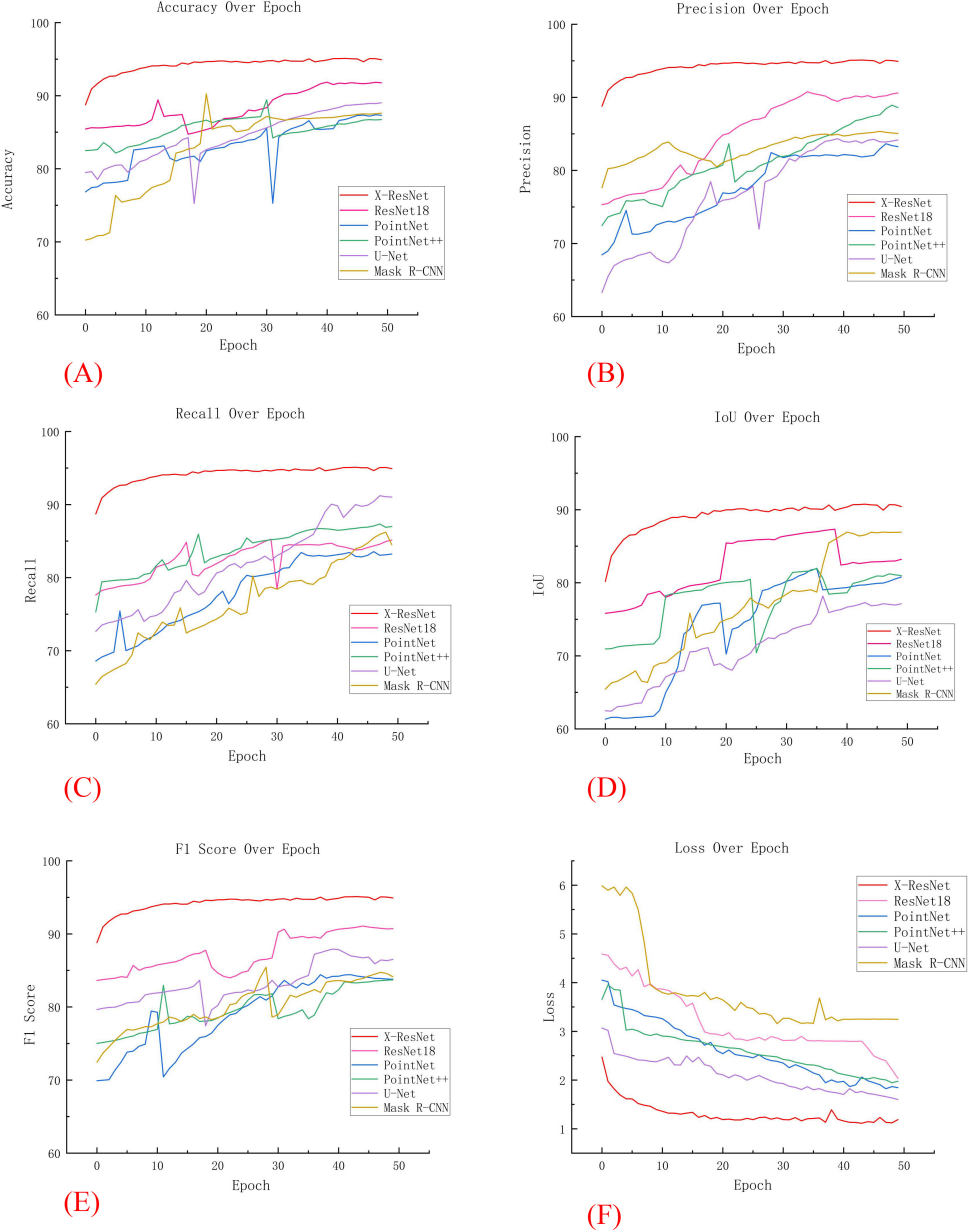
Comparison of various data in comparative experiments. **(A)** Accuracy; **(B)** Precision; **(C)** recall; **(D)** IoU; **(E)** F1 Score; **(F)** Loss.

In five additional comparative experiments, the models required a substantial number of parameters and exhibited high complexity. As the number of iterations increased, these models were prone to overfitting, resulting in significant fluctuations in various training metrics. In contrast, as illustrated in **Figure 5A**, the X-ResNet network model maintained a dynamic balance in accuracy as the number of iterations increased. Throughout the iterative process, the X-ResNet model achieved the highest accuracy without significant fluctuations, demonstrating excellent stability. The experimental results indicate that the X-ResNet network model exhibits superior performance across various metrics, enhancing training accuracy and yielding the best segmentation results.

### 3.2 Ablation experiment

To investigate the degree to which various modifications impact the training performance of the X-ResNet network model, this study conducted ablation experiments to validate their effectiveness. **Table 2** shows the performance comparison of network models trained on tomato plants. After integrating the bottleneck block into the ResNet18 network and comparing it with the traditional ResNet18 network, observed an increase of 1.41 in accuracy, 7.5 in recall, and 1.06 in F1-Score. However, after adding the downsampling operation to the ResNet18 network, there was an increase of 1.3 in accuracy, 7.42 in recall, and 1.74 in precision. This indicates that ResNet18, as the baseline model, will improve the training effect of the network model by introducing bottleneck blocks or downsampling operations.

**Table 2:** Compares and analyzes the performance of the ResNet18 network, X-ResNet network, “ResNet18 + Bottleneck Block” variant network, and “ResNet18 + Downsampling” variant network in the tomato plant image segmentation task.

Models	Accuracy(%)	Recall(%)	Precision(%)	Loss	F1-score (%)	IoU (%)
ResNet18	91.85	85.15	90.61	20.35	91.08	87.33
ResNet18+Custom convolution	91.72	84.39	87.76	1.722	91.84	89.06
ResNet18+Bottleneck Block	93.26	92.65	90.15	18.99	92.14	86.92
ResNet18+Downsampling	93.15	92.57	92.35	22.20	90.89	87.88
X-ResNet	95.11	95.11	95.04	11.13	95.10	90.76

**Figure 6:**
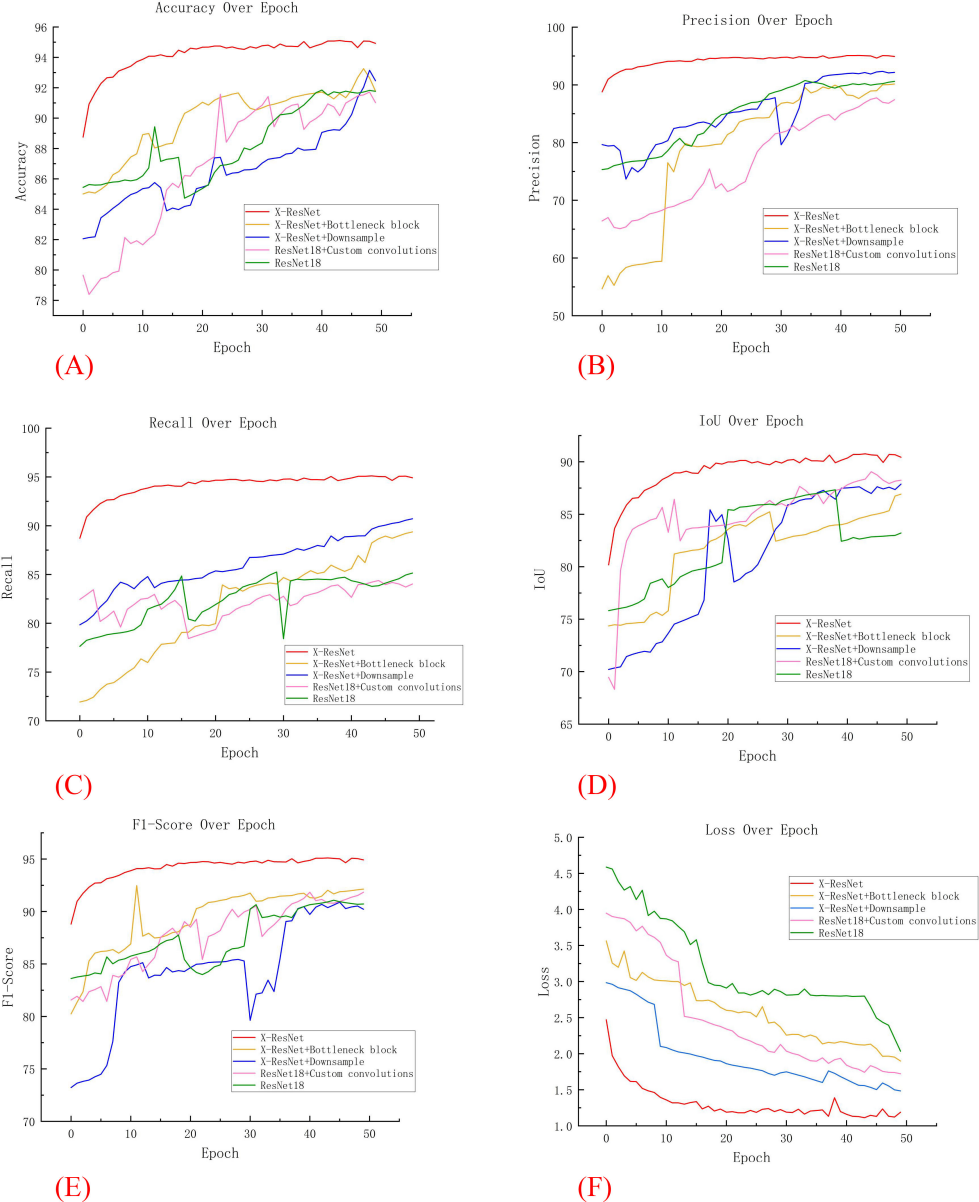
Comparison of various data in ablation experiments. **(A)** Accuracy; **(B)** Precision; **(C)** recall; **(D)** Iou; **(E)** F1 Score; **(F)** Loss.

**Figure 6** depicts the trends in evaluation metrics during the training process for these four models. Through comparative analysis, this research found that both the “ResNet18 + Downsampling” model and the “ResNet18 + Bottleneck Block” model demonstrated improved training effects after multiple iterations. In contrast, the traditional ResNet18 model exhibited significant fluctuations and poor training performance. At the same time, **Figure 6F** shows that with the increase of iteration ations of the X-ResNet network model, the loss function becomes smaller and smaller, and there is no large fluctuation. It can be concluded that variants of the ResNet18 network model improve segmentation accuracy by optimization adjustment, while the X-ResNet model achieves good performance on segmentation task by merging bottleneck blocks and downsampling operations.

### 3.3 Measurement results and analysis of phenotypic parameters

**Figure 7:**
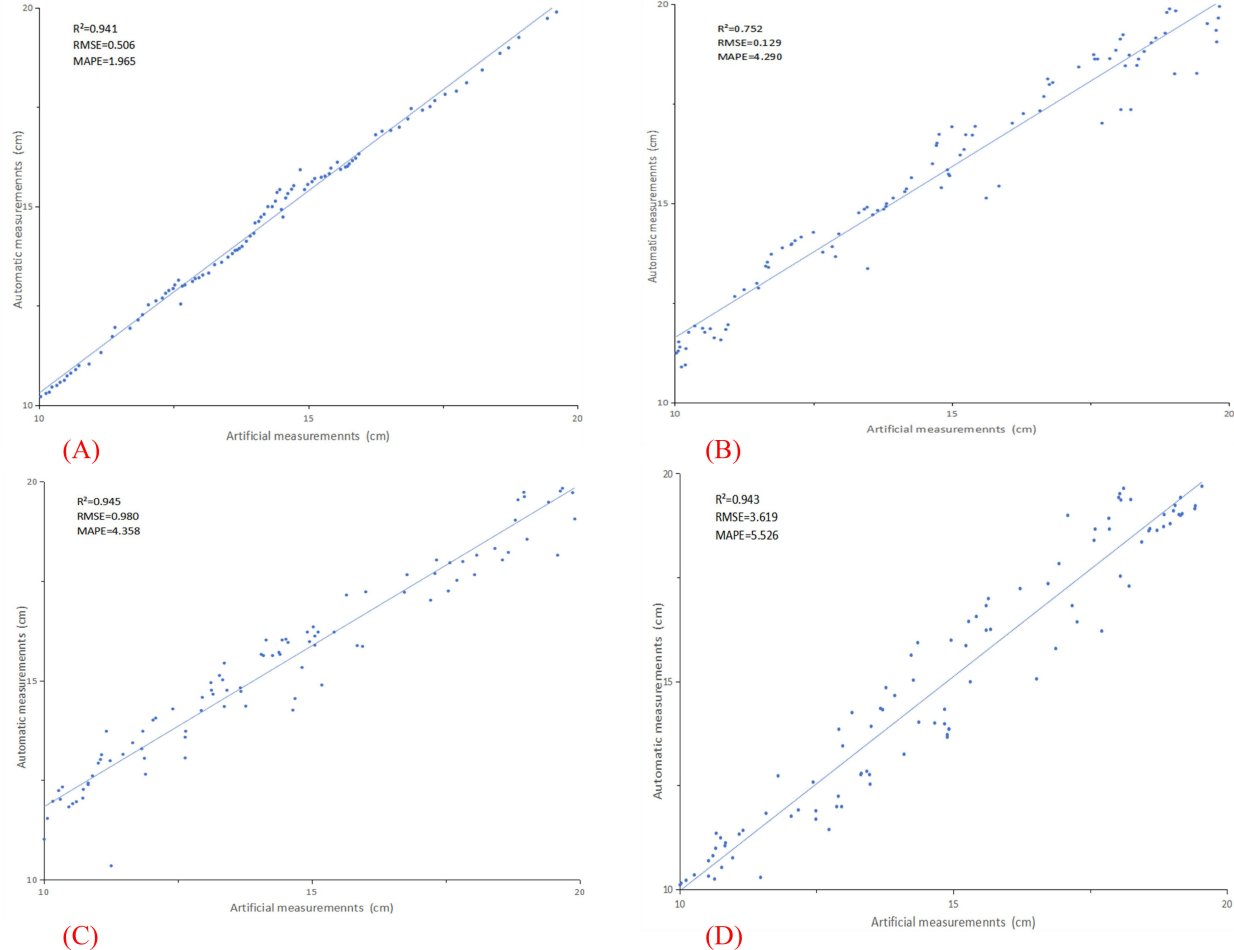
Displays the measurement results for four crucial plant phenotypic parameters. **(A)** plant height; **(B)** stem diameter; **(C)** leaf area; **(D)** leaf inclination angle.

In this study, 100 tomato plants were meticulously measured and analyzed, with a systematic comparison conducted between manual measurements and data extracted using advanced three-dimensional point cloud technology. **Figure 7** presents detailed measurement results for four key plant phenotypic parameters, specifically: (A) measurement analysis of plant height, with parameters of R²=0.941, RMSE=0.506, and MAPE=1.965; (B) measurement analysis of stem diameter, yielding parameters of R²=0.752, RMSE=0.129, and MAPE=4.290; (C) measurement analysis of leaf area, with parameters of R²=0.945, RMSE=0.980, and MAPE=4.358; and (D) measurement analysis of leaf inclination angle, yielding parameters of R²=0.943, RMSE=3.619, and MAPE=5.526. The experimental results demonstrate that through in-depth analysis of plant phenotypic parameters, the measured values obtained exhibit a high degree of correlation with the actual data, validating the accuracy and reliability of three-dimensional point cloud technology in plant phenotypic measurement.

## 4 Discussion

During the initial growth stage of tomato plants, the similarity in color characteristics between their leaves and stems poses a significant challenge to traditional image segmentation techniques, making it difficult to effectively differentiate targets with similar morphologies and colors. To address this issue, this experiment incorporated downsampling operations into the ResNet18 network model, significantly enhancing the model’s recognition accuracy for various plant organs (leaves and stems), and consequently improving the model’s training accuracy. This study enhances the model’s ability to capture local geometric information of point cloud data by incorporating curvature features and geometric features into the custom convolutional layer. This improvement is particularly evident in high-curvature regions such as stem and leaf segmentation, thereby boosting the model’s sensitivity to fine details and ultimately improving its training accuracy. Meanwhile, by introducing bottleneck blocks into the model, reduced the number of input and output channels, thereby decreasing the model’s complexity. In addition, after the convolutional layer, added a batch normalization layer, which accelerated the model’s convergence speed, improved its stability. The final experimental results demonstrate that the adoption of the X-ResNet network model for stem-leaf segmentation of tomato plants yields great segmentation results.

**Figure 8:**
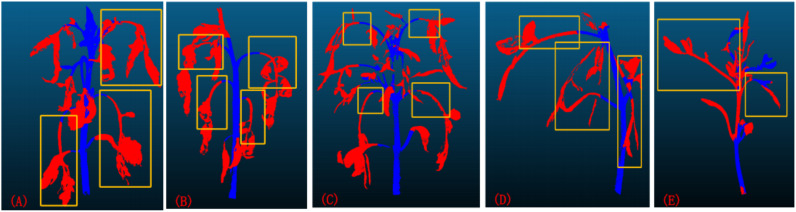
**(A–C)** The growth direction of the leaves is close to vertical; **(D, E)** The angle between the plant stem and the ground is too small.

Through comprehensive analysis of comparative experiments and ablation studies, confirmed the superior performance of the X-ResNet model in point cloud segmentation tasks. As shown in **Figures 8A to 8C**, when the leaves of tomato plants are excessively long and heavy, resulting in a nearly vertical growth direction, it adversely affects the training effectiveness of the model. Similarly, as illustrated in **Figures 8D, 8E**, when the angle between the plant stem and the ground is too small, it also leads to errors in the model’s identification of stems and leaves, thereby weakening the training performance. Despite the network model demonstrating good training results on the tomato dataset, continuous optimization and improvement are still required in subsequent research. When using a point cloud camera for image acquisition under strong or low light conditions, excessive or insufficient light intensity can lead to underexposure or overexposure. Additionally, unsuitable lighting conditions affect the reflectance and texture characteristics of plant surfaces, significantly impacting the accuracy of the camera’s 3D scanning. These factors can degrade the quality of point cloud data during acquisition, thereby compromising the reliability of the training dataset. This experiment was validated only during the vegetative growth stage of tomato plants. Future research will employ transfer learning to extend the X-ResNet network model to other crop varieties and systematically validate different growth stages of plants to enhance the model’s generalization ability. In smart greenhouse systems, this model will be integrated with low-power edge computing devices to enable real-time environmental parameter adjustment and growth monitoring based on plant growth status, thereby increasing crop yield and improving the precision of agricultural monitoring.

## 5 Conclusion

This paper proposes a lightweight deep learning-based network structure for high-precision segmentation of plant stems and leaves, which has been trained and validated on a tomato plant dataset. Experimental results demonstrate the model’s excellent segmentation performance. This method fully leverages point cloud data, significantly enhancing the model’s ability to capture spatial information, thereby optimizing the accuracy during model training. This method utilizes a convolutional layer that integrates curvature features and geometric features to process point cloud data, thereby enhancing the model’s capability to capture the characteristics of different organs of tomato plants and optimizing the accuracy during the model training process. This research incorporate bottleneck blocks into the traditional network architecture, effectively reducing the number of parameters and computations, and thus lowering the model’s complexity. Additionally, after each convolutional layer, this research thoughtfully add Batch Normalization (BN) layers, which significantly improve the training stability of the model. By introducing downsampling operations, this research effectively mitigate overfitting, thereby enhancing the model’s robustness to noise and training accuracy. The improved model is capable of performing more precise segmentation of plant parts. By calculating evaluation metrics such as accuracy and recall, found that the X-ResNet network model consistently exhibits good performance during training. In future research, this research will continue to explore the training effectiveness of this model on datasets of more plant species, aiming to continuously improve its generalization ability, thereby increasing crop yields in agricultural production.

## Data Availability

The datasets presented in this article are not readily available due to privacy concerns. Requests to access the datasets should be directed to Xinying Li, lixinying@mails.jlau.edu.cn.
